# Testosterone Deficiency Caused by Castration Modulates Mitochondrial Biogenesis Through the AR/PGC1α/TFAM Pathway

**DOI:** 10.3389/fgene.2019.00505

**Published:** 2019-05-29

**Authors:** Can Liu, Jideng Ma, Jinwei Zhang, Han Zhao, Yan Zhu, Jing Qi, Lingyan Liu, Li Zhu, Yanzhi Jiang, Guoqing Tang, Xuewei Li, Mingzhou Li

**Affiliations:** Farm Animal Genetic Resource Exploration and Innovation Key Laboratory of Sichuan Province, Sichuan Agricultural University, Chengdu, China

**Keywords:** mitochondrial biogenesis, testosterone, androgen receptor, TFAM, castration

## Abstract

Mammalian mitochondrial biogenesis is a complex process involving mitochondrial proliferation and differentiation. Mitochondrial DNA transcription factor A (*TFAM*), which encodes a major component of a protein-mitochondrial DNA (mtDNA) complex, is regulated by peroxisome proliferator-activated receptor γ coactivator 1α (PGC1α). Testosterone is the primary male sex hormone and plays an increasingly important role in mammalian development through its interaction with androgen receptor (AR). However, the function of *AR* in mitochondrial biogenesis induced by testosterone deficiency has not been investigated. Here, we explored the molecular mechanism underlying the effect of testosterone deficiency on mitochondrial biogenesis using a Yorkshire boar model. Testosterone deficiency caused by castration induced changes in mtDNA copy numbers in various tissues, and *AR* showed the opposite tendency to that of mtDNA copy number, particularly in adipose tissues and muscle tissues. In addition, castration weakened the correlation of *PGC1α* and mtDNA copy number, while *AR* and *TFAM* showed a relatively high correlation in both control and castrated pigs. Furthermore, luciferase assays revealed that *AR* binds to potential *AR* elements in the *TFAM* promoter to promote *TFAM* expression. Taken together, testosterone may be involved in the pathway linking PGC1α to mitochondrial biogenesis through the interaction between *AR* and *TFAM*.

## Introduction

As vital organelles in eukaryotic cells, mitochondria are involved in various biological processes, including cell energy metabolism (Aversa et al., 2017), calcium ion homeostasis (Detmer and Chan, 2007), and cell apoptosis (Teslaa et al., 2016). In mammals, the quantity, structure, and function of mitochondria can critically affect the development of various tissues and organs, especially those with higher energy metabolism, such as heart (Gustafsson and Gottlieb, 2008) and skeletal muscle (Wu et al., 2002). The two opposing processes, mitochondrial biogenesis and mitochondrial autophagy, finely influence the mitochondrial homeostasis, and an imbalanced response to either of two the processes results in functional deterioration of biological systems and promotes cell death (Palikaras and Tavernarakis, 2014). Mitochondrial biogenesis is a complex process involving mitochondrial proliferation and differentiation and is synergistically regulated by both mitochondrial and nuclear genomes (Attardi and Schatz, 1988). The PGC1 family plays a key role in inducing mitochondrial biogenesis and improving mitochondrial respiration (Lehman et al., 2000). As the master regulator of mitochondrial biogenesis, PGC1α can activate nuclear respiratory factors (*NRF-1*) and then upregulate the gene expression of nuclear *TFAM*, the first identified mitochondrial DNA (mtDNA) transcription factor that is essential for proper mtDNA copy number and transcription (Ekstrand et al., 2004; Clay Montier et al., 2009; Taherzadeh-Fard et al., 2011). Furthermore, recent studies have showed that Parkin and Pink1 proteins are involved in the mitochondrial autophagy induced by reduced membrane potential (Narendra et al., 2008, 2010). In addition, *Bnip3* (Lee et al., 2011; Hanna et al., 2012) and *ATG7* (Mortensen and Simon, 2010) are also closely related to mitochondrial autophagy. Accumulating evidence showed that mitochondrial dysfunction is closely related to human mtDNA-mutation diseases including skeletal muscle atrophy (Theilen et al., 2016), prostate cancer (Zhou et al., 2014), cardiovascular disease (Tsutsui et al., 2008) and breast cancer (Thyagarajan et al., 2013), which are associated with variations in mtDNA copy number. This suggests the crucial role of mitochondrial homoeostasis in maintaining a variety of normal physiological processes (Eskiocak et al., 2016). However, the tissue profile of mtDNA copy numbers and mitochondrial homoeostasis-associated gene expressions in mammals has not been well-studied.

Testosterone is the primary male sex hormone secreted by testis interstitial cells and has a wide variety of effects on sex differentiation (Isidori et al., 2005), fat deposition (Mammi et al., 2011; Kelly and Jones, 2013), muscle growth (Schaap et al., 2005), the cardiovascular system (Reckelhoff, 2005; Lopes et al., 2012), and the immune system (Trigunaite et al., 2015). In the cell cytoplasm, testosterone is often converted by 5α-reductase to the more active form, dihydrotestosterone (DHT). DHT efficiently binds to AR and triggers an AR conformational change, heat shock protein disaggregation and AR phosphorylation. AR then translocates to the nucleus, where it can recruit coactivators and transactivates testosterone-responsive genes by binding to androgen response elements (AREs) in the gene promoters (Shaffer et al., 2004; Burek et al., 2007). Although AR is widely expressed in mammalian cells and tissues, testosterone exerts its pleiotropic effects via AR-dependent or AR-independent mechanisms (Torres-Estay et al., 2017; Gaba et al., 2018). For example, testosterone prevents atherosclerosis through improving endothelial cell growth and survival via AR-independent mechanisms. In contrast, testosterone and DHT stimulate vascular smooth muscle cell proliferation via AR-independent and AR-dependent pathways (Nheu et al., 2011). Recent studies showed that testosterone can promote the mitochondrial biogenesis in skeletal muscle (Usui et al., 2014), and inhibit the proliferation of mitochondria in white adipocytes (Capllonchamer et al., 2014). Zawada et al. (2015) also indicated that the biogenesis of mitochondria might be regulated by sexual dimorphism, and further proved that testosterone and mitochondria are closely related. In addition, recent studies found that the knockdown of ATP1A1, an androgen-regulated gene, would induce mitochondrial dysfunction by disrupting ion homoeostasis, hence indicating the existence of the AR-mitochondria pathway (Jin et al., 2013; Eskiocak et al., 2016; Takase et al., 2017). Nonetheless, the potential relationship and underlying mechanisms between testosterone and mitochondrial homeostasis have not been fully illustrated.

In this study, we established a testosterone deficiency model in Yorkshire boars by prepubertal castration to research the effect and underlying molecular mechanism of testosterone on the *AR* distribution pattern and mitochondrial homoeostasis in various tissues. Our results suggested that testosterone might have pleiotropic effects on mitochondrial homoeostasis and the AR distribution pattern. These findings provide a foundation for further studying the correlation between testosterone and mtDNA copy number in different tissues and the effect of testosterone in the modulation of this link.

## Materials and Methods

### Animals and Tissue Collection

The experimental procedures used in this study were approved by the Institutional Animal Care and Use Committee of Sichuan Agricultural University (Approval No. DKY-S20153307, 15 November 2015). A total of twenty-four Yorkshire boars (including 12 pairs of full siblings) were used in this study. At the age of 7 days, both testicles of one piglet in each pair were removed by surgical castration under anesthesia (castrated group); the control piglet in each pair remained intact (control group). Animals were fed with free access to food and water. At the age of 10 months, all animals were humanely killed as necessary to ameliorate suffering and not fed the night before they were slaughtered. The phenotypic parameters of all animals (*n* = 24) in both group (control and castrated group) were determined, including body weight, serum testosterone level, and visceral indexes (i.e., the ratio of tissue weight/body weight). Next, four pigs of each group were randomly selected for tissue collection and subsequent assays. Adipose tissues (upper layer of backfat, inner layer of backfat, mesenteric adipose, intermuscular adipose, retroperitoneal adipose, greater omentum), muscle tissues (psoas major muscle, longissimus dorsi muscle, corpus linguae, left atrium, left ventricle), endocrine glands (adrenal gland, prostate, seminal vesicle, mammary gland), immunologic tissues (spleen, mesenteric lymph nodes, caecal submucosal lymphatic) and other major organs (liver, lung, kidney) were collected according to the guidelines for the care and use of experimental animals established by the Ministry of Agriculture of China. Tissues were then rapidly divided into small pieces, and partially frozen in liquid nitrogen and stored at -80°C until RNA and DNA extraction The remaining parts were stored with formalin fixation.

**FIGURE 1:**
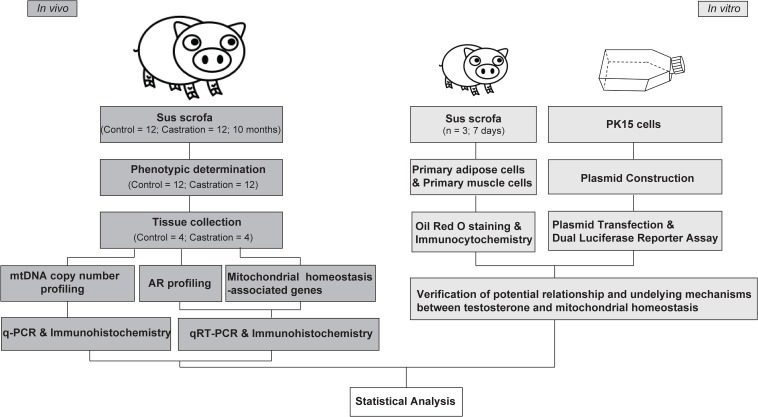
Experimental design overview.

Three healthy male Yorkshire piglets were selected for primary cell preparation. Animals were killed at the age of 7 days under isoflurane anesthesia, by artery bleeding from the exposed aorta, and then disinfected with 75% alcohol. The piglets were rapidly dissected. Subcutaneous adipose tissues (regio scapularis) and *longissimus dorsi* muscle tissues were sampled under aseptic condition and used immediately for primary cell isolation. The experimental design is shown in the Figure 1.

### Quantitative Real-Time RT-PCR (qRT-PCR)

Total RNA was extracted from tissues and cells using the RNAiso Plus reagent (TaKaRa, Tokyo, Japan) according to the manufacturer’s instructions. RNA concentration was detected using a NanoDrop^TM^ 2000 spectrophotometer (Thermo Scientific, Waltham, MA, United States). Complementary DNA (cDNA) was synthesized using the PrimeScript^TM^ RT reagent kit with gDNA Eraser (TaKaRa). cDNA was examined by qRT-PCR using the SYBR^®^Premix Ex Taq^TM^ II (Tli RNaseH Plus) (TaKaRa) with specific primers and the CFX384 Real-Time PCR Detection System (Bio-Rad, Hercules, CA, United States). Data were normalized by *GADPH* and *β-actin* mRNA expression for each sample. The relative expression of mRNAs was calculated using the comparative 2^-ΔΔCT^ method. Primer sequences used in this study are shown in Supplementary Table S1; all primers used in this study were synthesized by Tsingke (Chengdu, China).

### MtDNA Quantification

We evaluated the mtDNA content relative to nuclear DNA by qRT-PCR as previously described (Miller et al., 2003), using diluted total DNA as a template and primers for ATP synthase F0 subunit 6 (*ATP6*), NADH dehydrogenase subunit 1 (*ND1*) and cytochrome c oxidase subunit 1 (*COX1*) as mitochondrial genes and glucagon (*GCG*) as a nuclear gene. Twice the ratio of *ATP6*/*COX1*/*ND1* to single-copy nuclear gene number reflected the mtDNA amount per cell.

### Immunohistochemistry

Fixed tissues were dehydrated using a full-automatic dehydrator, paraffin embedded and then sectioned into 5-mm thick samples. First, the dewaxed sections were placed into the dyeing tank with 3% methanol hydrogen peroxide at room temperature for 10 min. The samples were rinsed with PBS three times for 5 min each. The slices were dipped into 0.01 M citrate buffer (pH 6.0) and then heated in the microwave until boiling for 5 min interval; the heating was repeated once more. After cooling, the slice was washed with PBS two times for 5 min each. The sections were then blocked with a blocking serum (ZLI-9021, ZSGB-BIO) at room temperature for 20 min. The sections were incubated with the AR (C-19) antibody (1:100; sc-815, Santa Cruz) at 4°C overnight and then with a secondary antibody (1:250; sp-9001, ZSGB-BIO) for 30 min at 37°C. Samples were rinsed with PBS three times for 5 min each and then processed with the Concentrated DAB kit (K135925C, ZSGB-BIO). Sections were then dehydrated in alcohol, cleared in xylene and mounted in synthetic resin. Mitochondria staining of paraffin tissue sections was carried out using the anti-prohibitin antibody mitochondrial marker (1:100; ab28172, Abcam). The mean density of immunohistochemical staining was measured using Image-Pro Plus (IPP). *n* = 4 per group; five technical replicates.

### Primary Cell Culture

Adipose tissues and muscle tissues samples were immersed in the PBS, supplemented with 100,000 U/L penicillin sodium and 100 mg/L streptomycin sulfate (Hyclone), washed three times and cut in small pieces with scissors, respectively. Adipose tissues were incubated with collagenase I in a shaking bath with 40 rpm speeds at 37°C for 1 h (muscle tissues were incubated for 1.5 h). After incubation, we neutralized the supernatant with an equivalent volume of complete medium that contained DMEM-F12 (Hyclone, Logan, UT, United States), then supplemented with 10% fetal bovine serum (GIBCO, Grand Island, NY, United States), 100,000 U/L penicillin, and 100 mg/L streptomycin (Hyclone). Subsequently, the suspensions were gently sieved through a cell strainer with 70 and 40 μm (Millipore, Billerica, MA, United States), centrifuged (1,000 rpm for 10 min) and discarded to remain the cell sedimentation. Afterward, the cells were washed twice with equivalent volume of PBS by centrifugation, resuspended in complete medium and seeded onto a 6-well culture plates (Corning).

The primary preadipocytes were cultured in DMEM-F12 (Hyclone, Logan, UT, United States) supplemented with 10% FBS and incubated at 37°C in a humid atmosphere of 5% CO_2_. At 2 days post-confluency (day 0), the medium was replaced with complete medium containing 500 μmol/L 3-isobutyl-1-methylxanthine (Sigma-Aldrich), 1 μmol/L dexamethasone (Sigma-Aldrich) and 1 ug/mL insulin (Sigma-Aldrich). After 2 days (day 2), the medium was changed to the complete medium containing 1 μg/mL insulin. After another 2 days (day 4), the medium was changed to the complete medium, which was then replaced every 2 days. The testosterone (10^-7^ mol/L) and EPI-001 (10^-4^mol/L) were added to the medium every change after day 0.

The primary myoblasts were cultured in DMEM-F12 supplemented with 10% FBS and incubated at 37°C in a humid atmosphere of 5% CO_2_. For differentiation, when cells reached 90% confluence, the medium was exchanged for a differentiation medium containing 2% horse serum (GIBCO, Grand Island, NY, United States). Testosterone (10^-7^ mol/L) and EPI-001 (10^-4^mol/L) were soluble in differentiation medium. The medium was changed every other day.

### Oil Red O Staining

When differentiated at day 9, adipocytes were stained with Oil Red O (Sigma-Aldrich) to detect lipids. After removing the differentiation medium, cells were gently washed three times with PBS, and fixed in 10% formalin for 30 min. The fixed cells were stained with 0.5% Oil Red O for 1 h at room temperature. After cells were washed three times with PBS again, images were captured by an Olympus IX53 microscope (Olympus, Tokyo, Japan). To measure triglyceride contents, stained cells were eluted with isopropanol for 20 min, and the OD values were detected with a spectrophotometer at a wavelength of 510 nm.

### Immunocytochemistry

The differentiated myoblasts were rinsed in PBS three times and washed in 0.3% Triton X-100 for 30 min, followed by blocking at room temperature for 1 h. These cells were incubated in anti-fast-MyHC (ab51263, Abcam) at 4°C overnight. The next day, the cells were incubated with Alexa Fluor^®^488 secondary antibody (ab150113; Abcam) for 1 h, and the cell nucleuses were stained by DAPI (Beyotime, Shanghai, China). Finally, the cells was imaged using Olympus IX53 microscope (Olympus, Tokyo, Japan) with cell Sens Standard software (v1.16, Olympus Instruments, Tokyo, Japan). Up to six fields of view were captured from each group.

### Plasmid Construction

The potential AR binding sites in the *TFAM* promoter were predicted by JASPAR (Portalescasamar et al., 2010) and PROMO 3.0 (Messeguer et al., 2002). The sequence of the *TFAM* promoter containing the specific ARE binding sites (wild-type or mutant) was synthesized from TSINGKE (Chengdu, China), cleaved using Sac I/Xho I, and then inserted into the pGL3-basic reporter vector.

### Plasmid Transfection and Dual Luciferase Reporter Assay

PK15 cells were seeded in 48-well plates. These cells, with 70–80% confluence, were transfected using Lipofectamine TM 3000 (Invitrogen, Carlsbad, CA, United States). Each well was transfected with 0.2 μg recombinant pGL3 vector and 0.02 μg internal control vector pRL-TK DNA using Lipofectamine TM 3000 (Invitrogen, Grand Island, NY, United States). Cells were collected 48 h after transfection and dual luciferase activity was measured using the Dual-Luciferase Reporter Assay System kit (Promega, Madison, WI, United States), according to the manufacturer’s instructions.

### Statistical Analysis

Statistical comparisons were performed with Student’s *t*-test and one-way analysis of variance with Tukey’s *post hoc* test to evaluate the statistical significance for comparisons of two groups and multiple groups, respectively, using SPSS 19.0 software (SPSS, Inc., Chicago, IL, United States). Spearman’s rank correlation analysis was used for detecting the relationship between the mitochondrial homoeostasis-associated gene mRNA expression and the mtDNA copy number. Values of *P* < 0.05 were considered statistically significant. The data were expressed as means ± SD.

## Results and Discussion

### Establishment of a Testosterone Deficiency Model by Castration in Yorkshire Boars

**FIGURE 2:**
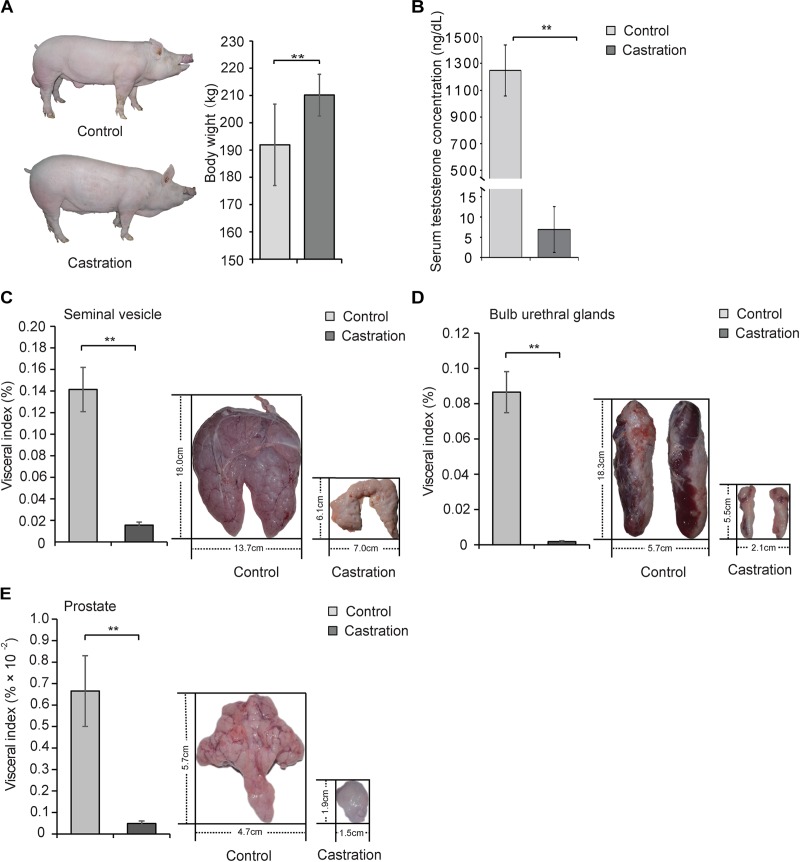
Prepubertal castration induced the decrease of serum testosterone and dysplasia of the accessory glands in adult Yorkshire boars. Castrated and control Yorkshire boars were established at 7 days old as described in Section “Materials and Methods” and the following parameters were determined in adult pigs (10 months old): **(A)** Body weight of control and castration. **(B)** Serum testosterone level and the visceral index (left panel) and size (right panel) of **(C)** seminal vesicle, **(D)** bulb urethral glands, and **(E)** prostate tissue. All data are expressed as means ± SD, *n* = 12 in each group. Student’s *t*-test, ^∗∗^*P* < 0.01.

Testis is an important site for spermatogenesis and secretion of androgen in male mammals (Wang et al., 2009). Testosterone deficiency caused by surgery or drug castration is widely used in animal production (Traish et al., 2003; Bretschneider, 2005) and serves as the main strategy for the clinical treatment of prostate cancer (Snyder et al., 2016). In this study, we established a testosterone deficiency model by prepubertal castration of Yorkshire boars at age of 7 days to investigate the effects of testosterone on mitochondrial homoeostasis. Interestingly, the body weight of the castrated pig obviously increased (Figure 2A). As shown in Figure 2B, prepubertal castration markedly resulted in a decrease of serum testosterone compared with the control in adult Yorkshire boars (10 months old) (*P* < 0.01). Castration also caused severe dysplasia of the accessory glands, including seminal vesicles [visceral index: 14.15 ± 2.06 versus 1.56 ± 0.30 (% × 10^-2^), *P* < 0.01; length: 18.01 ± 1.06 versus 6.05 ± 0.70 cm, *P* < 0.001; width: 13.73 ± 0.86 versus 6.95 ± 0.63 cm, *P* < 0.001], bulb urethral glands [visceral index: 8.66 ± 1.16 versus 0.18 ± 0.05 (% × 10^-2^), *P* < 0.01; length: 18.29 ± 1.25 versus 5.50 ± 0.66 cm, *P* < 0.001; width: 5.66 ± 0.75 versus 2.12 ± 0.23 cm, *P* < 0.01] and prostate [visceral index: 0.67 ± 0.16 versus 0.05 ± 0.01 (% × 10^-2^), *P* < 0.01; length: 5.67 ± 0.35 versus 1.92 ± 0.18 cm, *P* < 0.001; width: 4.71 ± 0.59 versus 1.48 ± 0.18 cm, *P* < 0.001] (Figure 2C–E). These results were consistent with previous reports that testosterone deficiency caused by castration led to reproductive system maldevelopment (O’Hara et al., 2011) and indicated the successful establishment of a testosterone deficiency model in Yorkshire boars.

### Testosterone Deficiency Alters mtDNA Copy Number Pattern in Various Tissues

**FIGURE 3:**
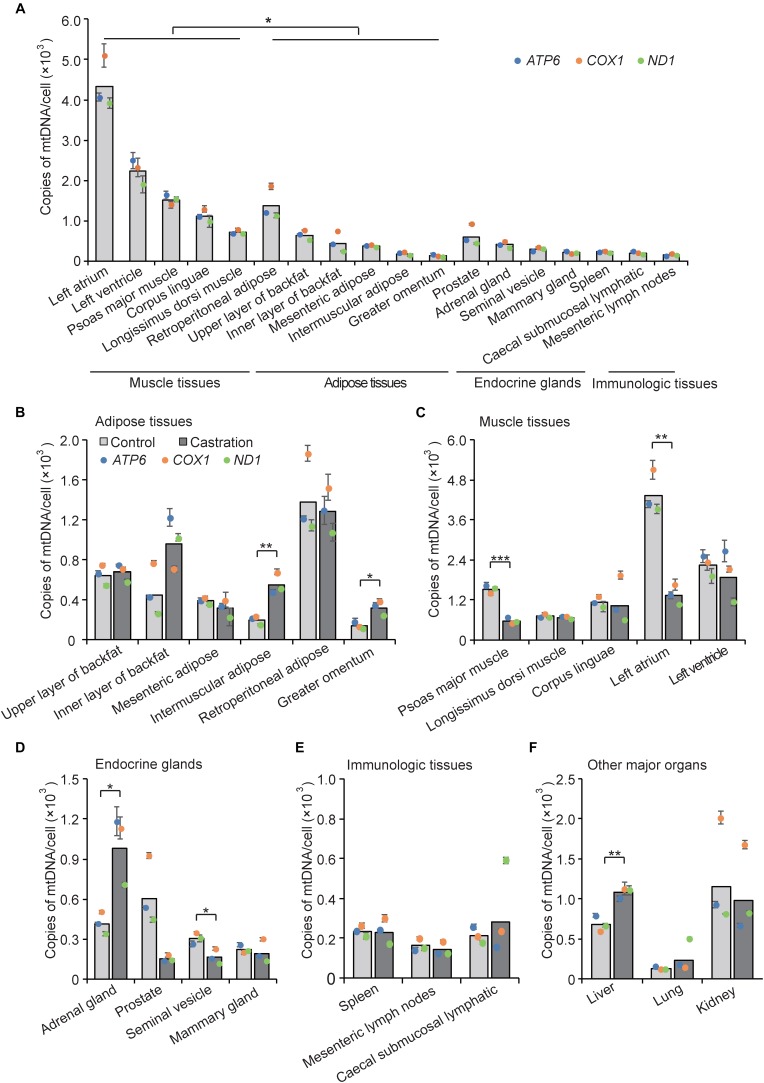
mtDNA copy number was determined by qRT-PCR analysis of three mitochondria-specific genes (*ATP6, COX1*, and *ND1*). Twice the ratio of *ATP6/COX1/ND1* to single-copy nuclear gene number reflected the relative mtDNA content, and the solid bars represent mean values of mtDNA contents individually calculated by *ATP6, COX1*, and *ND1*. **(A)** Tissue profiling of mtDNA copy numbers in five categories of tissues in wild-type pigs. mtDNA copy numbers per cell in adipose tissues **(B)**, muscle tissues **(C)**, endocrine glands **(D)**, immunologic tissues **(E)**, and other major organs **(F)** in control and castrated pigs. All data are expressed as means ± SD, *n* = 4 in each group. Comparison between control and castration groups was performed by Student’s *t*-test, ^∗^*P* < 0.05, ^∗∗^*P* < 0.01, ^∗∗∗^*P* < 0.001.

The amount of mtDNA is closely related to the level of energy metabolism in almost all cells and shows different distribution patterns in various tissues. For example, heart and skeletal muscle show a high energy demand, and each of these cells contain on average 10s of 1000s of mtDNA copies (Miller et al., 2003); in contrast, only a few 100 mtDNA copies are present in skin cells (García-Rodríguez, 2007). To examine the tissue expression distribution of mtDNA copy numbers in wild-type pigs, we determined mtDNA copy number as described in Section “Materials and Methods” by performing qRT-PCR of three mitochondria-specific genes (*ATP6, ND1*, and *COX1*) in five categories of tissue types, including adipose tissues, muscle tissues, endocrine glands, immunologic tissues, and other major organs. As shown in Figure 3A, we found that mtDNA copy numbers in muscle tissues were higher than that of adipose tissues (*P* < 0.05), followed by endocrine glands and immunologic tissues, indicating that muscle tissues with higher energy metabolism require more mtDNA copy numbers. Interestingly, adipose tissues showed similar mtDNA copy numbers as endocrine glands, which is in line with recent evidence that adipose tissues have endocrine function and can secrete numerous bioactive factors, termed adipokines (Kershaw and Flier, 2004; Waki and Tontonoz, 2007; Wozniak et al., 2009). Additionally, we also observed differential expression of mtDNA copy numbers within each tissue type, which indicated a tissue-specific distribution pattern of mtDNA copy number.

We next examined mtDNA copy numbers in the testosterone deficiency pigs and found that castration resulted in large differences of mtDNA copy numbers in various tissues compared with the control pigs. MtDNA copy numbers were increased in adipose tissues in castrated pigs, especially in intermuscular adipose (fold-change = 2.84; *P* < 0.01) and greater omentum (fold-change = 2.29; *P* < 0.05), whereas that of mesenteric adipose and retroperitoneal adipose showed the opposite trend indistinctively (fold-change = 0.80 and fold-change = 0.93, respectively; both *P* > 0.05) (Figure 3B). Castration caused a decrease of mtDNA copy numbers in muscle tissues especially psoas major muscle (fold-change = 0.37; *P* < 0.001) and left atrium (fold-change = 0.31; *P* < 0.01) (Figure 3C). These results were consistent with studies showing that testosterone increased the protein expression level of PGC1α and promoted mitochondrial biogenesis in skeletal muscle (Usui et al., 2014), while it inhibited the proliferation of mitochondria in white adipocytes *in vitro* (Capllonchamer et al., 2014). The mtDNA copy numbers in some endocrine glands were also lower in the castration group than the control, with a significant decrease in seminal vesicle (fold-change = 0.54; *P* < 0.05) and a decreasing trend in prostate and mammary gland. Notably, adrenal glands (fold-change = 2.37; *P* < 0.05) showed a marked increase of mtDNA copy number after castration (Figure 3D), which might suggest that castration induced compensatory secretion of testosterone in adrenal gland, accompanied with the increase of mtDNA copy numbers, to ensure energy supply. Moreover, there was no obvious change in mtDNA copy numbers between castration and control pigs in the immunologic tissues (Figure 3E), suggesting that the immunologic tissues may employ some compensatory mechanism to maintain mitochondrial function (Walker et al., 2014) under castration treatment. Interestingly, mtDNA copy number in liver showed a significant increase in the castration group (fold-change = 1.59; *P* < 0.01) (Figure 3F). Mitochondria play an important role in lipid oxidation in hepatocytes, and a previous study showed that the accumulation of oxidative damage caused by castration triggers a compensatory increase of mtDNA copy number (Kamfar et al., 2016). Taken together, the tissue-specific pattern of mtDNA copy numbers shown by these results implied that tissues with high energy demand are often associated with higher mtDNA copy numbers to coordinate the energy metabolism of the entire body, and testosterone modulated the tissue-specific distribution of mtDNA copy numbers.

**FIGURE 4:**
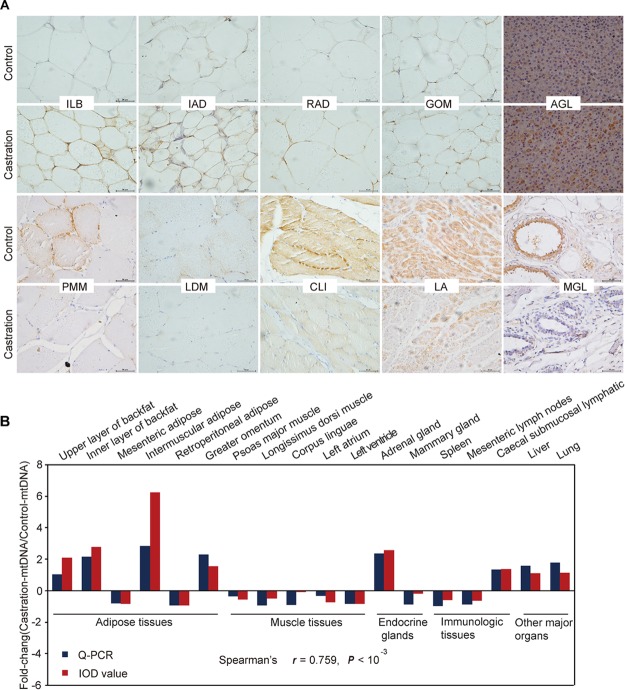
Immunohistochemical and correlation analysis between mitochondrial staining and qRT-PCR results of mtDNA. **(A)** Immunohistochemical analysis for 10 representative tissues using an anti-prohibitin antibody. Scale bars: 50 μm; 400× magnification. ILB, inner layer of backfat; IAD, intermuscular adipose; RAD, retroperitoneal adipose; GOM, greater omentum; AGL, adrenal gland; PMM, psoas major muscle; LDM, longissimus dorsi muscle; CLI, corpus linguae; LA, left atrium; MGL, mammary gland. **(B)** Fold change of mtDNA expression in tissues based on Spearman’s rank correlation. The relative expression levels were measured by qRT-PCR and IOD. *n* = 4 in each group.

We also performed immunohistochemistry to assess mtDNA localization and quantity using a specific anti-prohibitin antibody (a specific mitochondrial marker) for 18 representative tissues (Figure 4A and Supplementary Figure S1). We then analyzed the integrated optical density (IOD) (see more details in Supplementary Table S2) and the results showed a high correlation between mitochondrial staining and qRT-PCR results (Spearman’s *r* = 0.759; *P* < 0.001) (Figure 4B).

### Comprehensive Effect of Testosterone Deficiency on Mitochondrial Biogenesis

**FIGURE 5:**
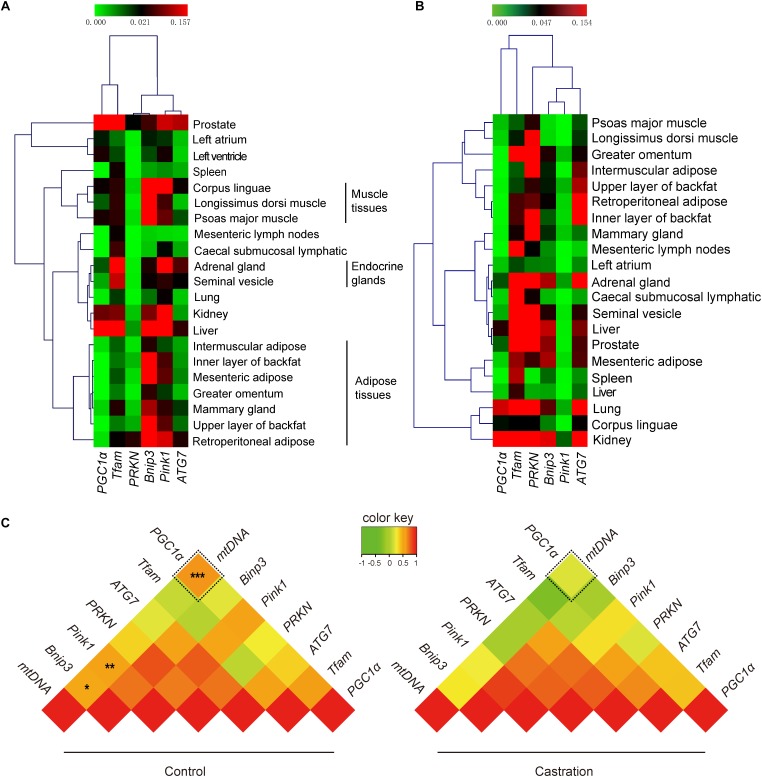
Tissue expression profiling of mitochondrial homeostasis-associated genes. The mRNA expression of *PGC1a, TFAM, ATG7, PRKN, Pink1*, and *Bnip3* and mtDNA copy numbers in various tissues. Hierarchical clustering analysis for the mRNA expression of mitochondrial biogenesis-associated genes in 21 tissues in **(A)** control pigs and **(B)** castrated pigs. **(C)** Correlation analysis of mitochondrial biogenesis and mitochondrial autophagy-associated genes with mtDNA copy number. Values represent the correlation coefficient (*r*) based on Spearman’s rank correlation analysis. All data are expressed as means ± SD, *n* = 4 in each group. Tukey’s *post hoc* test, ^∗^*P* < 0.05, ^∗^*P* < 0.01, ^∗∗∗^*P* < 0.001.

Our results showed that testosterone deficiency induced dramatic changes in mtDNA copy numbers in different tissues. mtDNA copy number is regulated by many factors, including mitochondrial biogenesis and mitochondrial autophagy (Gaziev et al., 2014; Santos et al., 2014). The dynamic balance of mitochondrial biogenesis and mitochondrial autophagy together determines the number of mitochondria (Santos et al., 2014). Thus, we next evaluated the expression of mitochondrial biogenesis-associated genes (*PGC1α* and *TFAM*) and mitochondrial autophagy-associated genes (*PRKN, Bnip3, Pink1*, and *ATG7*) using qRT-PCR analysis. Hierarchical clustering based on the expression profiles of these genes, that were co-expressed in all tissues of control pigs, showed that the six adipose tissues were tightly clustered into a subgroup and were clearly distinct from the three muscle tissues (Figure 5A). This significant distance in hierarchical clustering might reflect, at least in part, the physiological differences between adipose tissues and muscle tissues, for example, their functional and metabolic differences (Gondret et al., 2008). Interestingly, the mammary gland was clustered into the cluster of adipose tissues, which might attribute to the large amount of adipose tissues in the mammary glands (Santander et al., 2015). However, the expression pattern of mitochondrial homeostasis-associated genes was disrupted after castration (Figure 5B), which indicated that castration-induced testosterone deficiency dramatically altered the patterns of mtDNA copy number by modulating the mitochondrial homeostasis.

We next performed Spearman’s rank correlation analysis between mtDNA copy numbers and mitochondrial homeostasis-associated genes to further investigate the mechanism as to how the testosterone deficiency affects mitochondrial homeostasis. As shown in Figure 5C, mtDNA copy numbers significantly correlated with *PGC1α* (*r* = 0.636; *P <* 0.001), *Pink1* (*r* = 0.531; *P <* 0. 01) and *Bnip3* (*r* = 0.487; *P <* 0.05) in control pigs, whereas this correlation was significantly weakened after castration (*PGC1α, r* = 0.200, *P* > 0.05; *Pink1, r* = 0.266, *P* > 0.05; *Bnip3, r* = 0.288, *P* > 0.05). Notably, the fold change in correlation coefficient of *PGC1α* (fold-change = 3.18) was higher than that of *Pink1* (fold-change = 2.00) and *Bnip3* (fold-change = 1.69), which indicated that testosterone deficiency more significantly weakened the effects of *PGC1α* on mitochondrial biogenesis (see more details in Supplementary Table S3). Together, these results suggested that testosterone deficiency affected the mtDNA copy numbers by modulating mitochondrial homeostasis-associated genes, particularly in mitochondrial biogenesis, which may be through weakening the links between *PGC1a* and mtDNA copy numbers.

### The Potential Effect of AR on Mitochondrial Biogenesis

**FIGURE 6:**
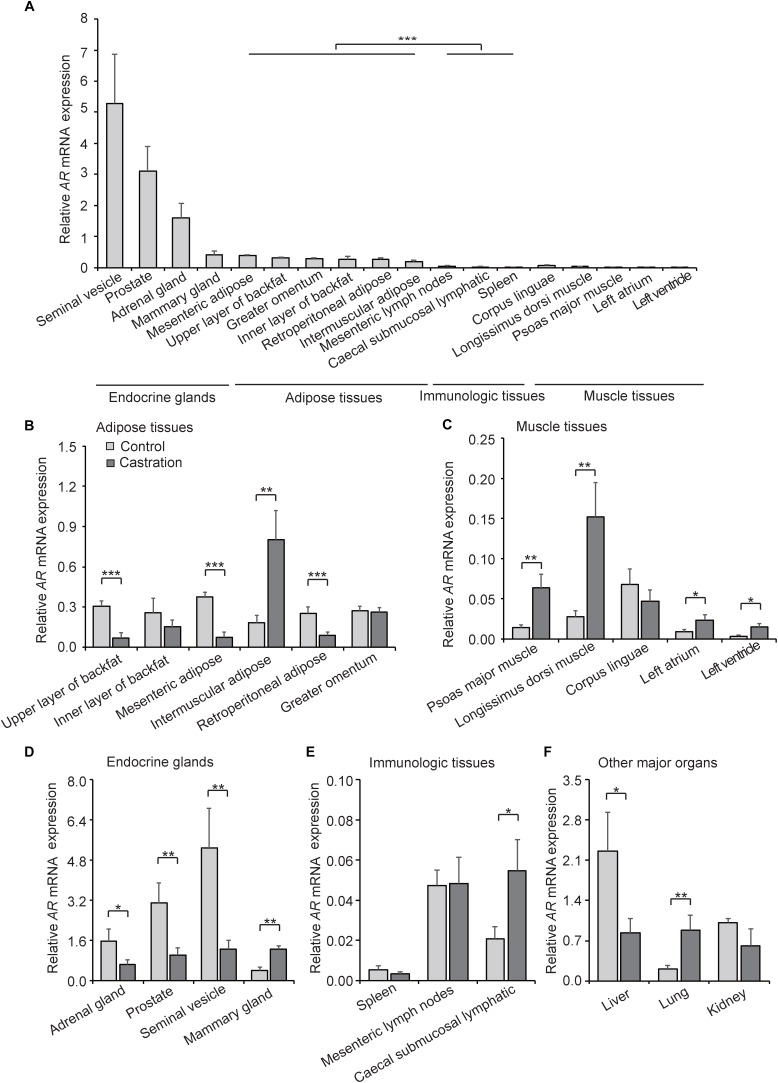
*AR* expression in various tissues. **(A)** Tissue expression profiling of *AR* in tissues from wild-type pigs. **(B)** *AR* mRNA expression in adipose tissues in control and castration pigs. **(C)** *AR* mRNA expression of muscle tissues in control and castration pigs. **(D)** *AR* mRNA expression of endocrine glands in control and castration pigs. **(E)** *AR* mRNA expression of immunologic tissues in control and castration pigs. **(F)** *AR* mRNA expression of other major organs in control and castration pigs. All data are expressed as means ± SD, *n* = 4 in each group. Student’s *t*-test,^∗^*P* < 0.05, ^∗∗^*P* < 0.01, ^∗∗∗^*P* < 0.001.

AR is a DNA-binding transcription factor which can be activated by binding androgenic hormones and is widely distributed in many types of cells. Testosterone exerts its physiological effects mainly through an AR-dependent process. Given that castration-induced testosterone deficiency resulted in the change of correlation between *PGC1a* and mtDNA copy number, we considered that AR might mediate this process. To examine this possibility, we first investigated the expression pattern of *AR* in various tissues in control pigs. As shown in Figure 6A, *AR* was highly expressed in many endocrine glands, especially in the gonads, followed by adipose tissues, and was relatively low in muscle tissues and immunologic tissues; these results indicated tissue-specific expression of *AR*. These results suggested that testosterone plays a crucial role in endocrine glands development and maturity, which is compatible with the higher expression of *AR* observed in our results.

We next compared the expression pattern of *AR* in the castration group with the control. An overall decrease of *AR* expression in adipose tissues in the castration pigs was detected, especially in the upper layer of backfat (fold-change = 0.22; *P* < 0.01), mesenteric adipose (fold-change = 0.19; *P* < 0.01) and retroperitoneal adipose (fold-change = 0.35; *P* < 0.01), whereas the *AR* expression of intermuscular adipose increased significantly after castration (fold-change = 4.40; *P* < 0.01) (Figure 6B). In muscle tissues, the expression of *AR* was higher in castration pigs than the control, including psoas major muscle (fold-change = 4.51; *P* < 0.01), longissimus dorsi muscle (fold-change = 5.52; *P* < 0.01), left atrium (fold-change = 2.72; *P* < 0.05) and left ventricle (fold-change = 4.08; *P* < 0.05) (Figure 6C). Moreover, the expression levels of *AR* in endocrine glands, including adrenal gland (fold-change = 0.41; *P* < 0.05), prostate (fold-change = 0.33; *P* < 0.01) and seminal vesicle (fold-change = 0.24; *P* < 0.01), were lower in castration pigs compared with the control. On the contrary, the mammary gland (fold-change = 3.08; *P* < 0.01) appeared to increase significantly in the castration group (Figure 6D). Previous studies showed that the testosterone reduction leads to gynecomastia (Sansone et al., 2017) and AR-mediated androgen actions play an important role in mammary physiology (Pakula et al., 2017), thus castration causes the pathological upregulation of *AR*. In our study, *AR* expression of caecal submucosal lymphatic in castration pigs (fold-change = 2.65; *P* < 0.05) increased significantly compared with the control (Figure 6E). Intriguingly, among other major organs, the *AR* level in liver was significantly decreased in the castration group (fold-change = 0.37; *P* < 0.05) along with an opposite trend in lung (fold-change = 4.20; *P* < 0.01) (Figure 6F). These results suggested that castration-induced testosterone deficiency altered the tissue-specific pattern of *AR* expression, and the effects of testosterone deficiency on *AR* expression showed the opposite tendency compared with mtDNA copy number, particularly in adipose tissues and muscle tissues.

*TFAM* is a crucial factor to maintain stability and transcription. TFAM participates in the regulation of mtDNA copy number and transcriptional activity, which are key regulators of mitochondrial content and functional capacity. Studies showed that PGC1α is indirectly involved in regulating mitochondrial biogenesis through increased expression of *TFAM*, which involves coactivation by NRF-1 (Taherzadeh-Fard et al., 2011). Thus, we performed Spearman’s rank correlation coefficient on *AR* and *TFAM* expression. Correlation analysis demonstrated that *AR* and *TFAM* were significantly correlated both in the control group (*r* = 0.388; *P <* 0.05) and the castration group (*r* = 0.390; *P <* 0.05) (Supplementary Table S4). Our results showed that *AR* might be involved in mitochondria biogenesis through a relationship with *TFAM*.

**FIGURE 7:**
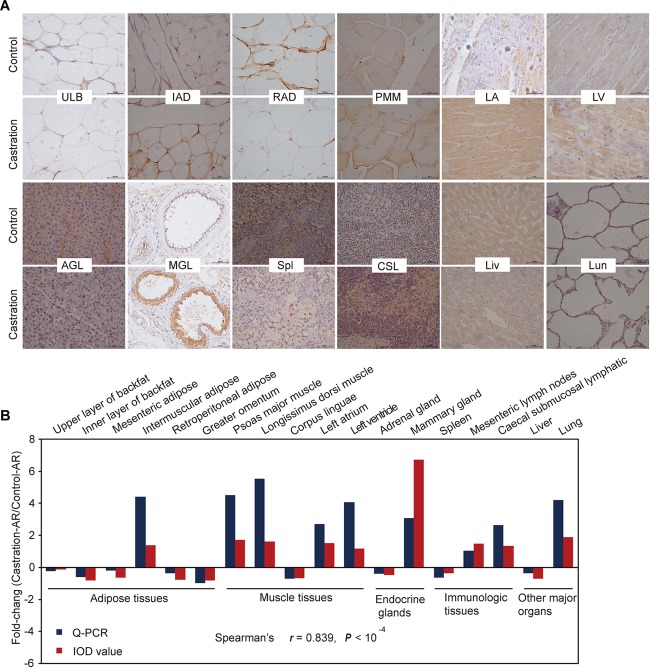
Immunohistochemical and correlation analysis between immunohistochemical staining and qRT-PCR results of AR. **(A)** Immunohistochemical analysis for 12 representative tissues using an AR (C-19) antibody. Scale bars: 50 μm; 400× magnification. ULB, upper layer of backfat; IAD, intermuscular adipose; RAD, retroperitoneal adipose; PMM, psoas major muscle; LA, left atrium; LV, left ventricle; AGL, adrenal gland; MGL, mammary gland; Spl, spleen; CSL, caecal submucosal lymphatic; Lun, lung; Liv, liver. **(B)** Fold change of AR mRNA expression in all tissues based on Spearman’s rank correlation. The relative expression levels were measured by qRT-PCR and IOD. *n* = 4 in each group.

We also performed immunohistochemistry analysis of AR for 18 representative tissues to verify the qRT-PCR results (Figure 7A and Supplementary Figure S2; see more details in Supplementary Table S5). The results from both these methods were highly correlated regarding AR expression (Spearman’s *r* = 0.839; *P* < 10^-4^) (Figure 7B).

### Validation of the Potential Correlation of AR and TFAM

**FIGURE 8:**
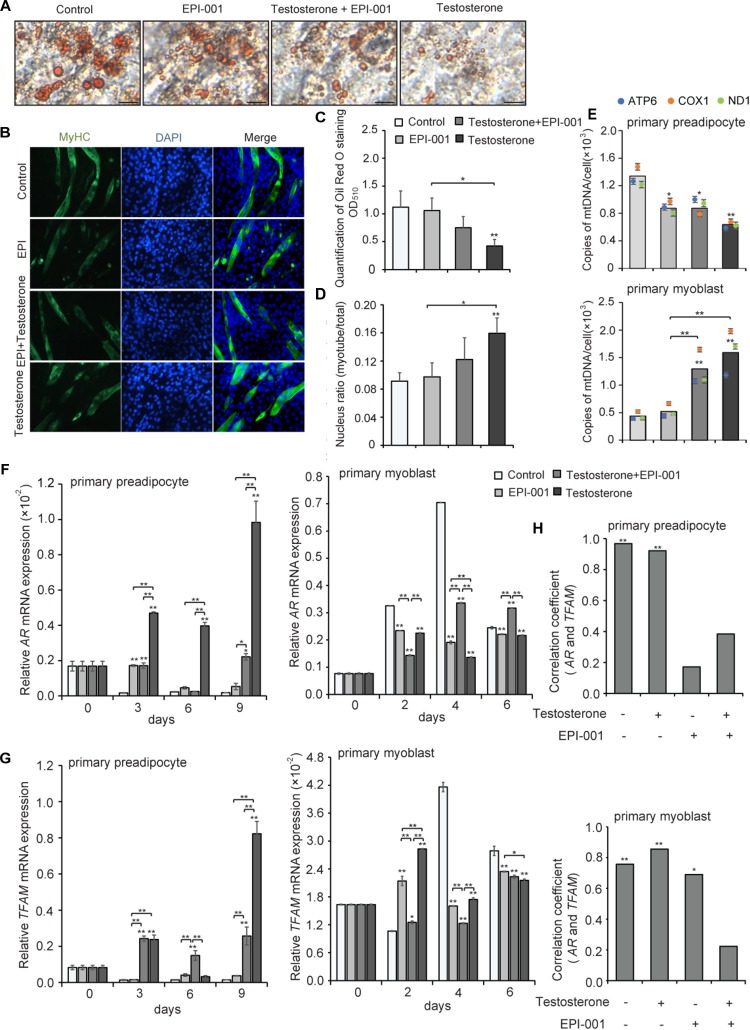
Effect of testosterone on pig primary cells during differentiation. **(A)** Oil Red O staining of adipocytes after treatment with testosterone (10^-7^ mol/L) and EPI-001 (10^-4^ mol/L) after differentiation for 9 day (scale bar = 50 μm). **(B)** Myotubes were stained with anti-MyHC antibody and the fusion index was calculated. Images were obtained by fluorescence microscopy on the 6th day of myoblast differentiation. **(C)** Quantification of Oil Red O staining. **(D)** Fusion index of myotube was calculated. **(E)** mtDNA copy number analysis of adipogenesis and myogenesis in four treatment groups. Twice the ratio of *ATP6/COX1/ND1* to single-copy nuclear gene number reflected the relative mtDNA content, and the solid bars represent mean values of mtDNA contents individually calculated by *ATP6, COX1*, and *ND1*. **(F)** *AR* and **(G)** *TFAM* expression of adipogenesis and myogenesis. **(H)** Correlation analysis of *AR* and *TFAM* based on a Pearson correlation coefficient test during adipogenesis and myogenesis, treated with 10^-7^ mol/L testosterone (T), 10^-4^ mol/L EPI-001 (EPI), and 10^-7^ mol/L testosterone + 10^-4^ mol/L EPI-001 (T+EPI-001). All data are expressed as means ± SD. Tukey’s *post hoc* test. ^∗^*P* < 0.05, ^∗∗^*P* < 0.01 compared with the control (on the bars) or between the indicated groups.

To further verify the correlation of *AR* and *TFAM*, pig primary preadipocytes and myoblasts were differentiated and cultured with or without testosterone during the entire differentiation process. EPI-001, as a small molecule, can inhibit transactivation of the AR N-terminal domain (NTD), that is essential for the activity to the ligand-bound AR (Andersen et al., 2010). We examined four treatment groups: control group, testosterone group (10^-7^ mol/L testosterone), testosterone + EPI group (10^-7^ mol/L testosterone, 10^-4^ mol/L EPI-001), and EPI group (10^-4^ mol/L EPI-001). After fully differentiating primary adipocytes at day 9, there was a significant difference in staining by Oil Red O among these different groups (Figure 8A). Meanwhile, myotubes and nuclear ratio were significantly increased upon treatment with testosterone (Figure 8B). Quantitation by extracting the OD values revealed that testosterone evidently inhibited the differentiation of preadipocytes in testosterone group compared with the control (*P* < 0.01), followed by testosterone + EPI-001 (*P* < 0.05) (Figure 8C). Conversely, the testosterone notably enhanced myotubes and nuclear ratio compared with the control (*P* < 0.01) and EPI-001 group (*P* < 0.05) (Figure 8D). We also found that testosterone could inhibit the proliferation of mitochondria in adipocytes (testosterone group versus control group, *P* < 0.01), and increase the mtDNA copy numbers in myotubes (testosterone group versus control group, *P* < 0.01), which was consistent with previous studies (Figure 8E).

Furthermore, we found that, during differentiation of primary preadipocytes, testosterone treatment enhanced relative expression levels of *AR* mRNA compared with the control at 3, 6, and 9 days (*P* < 0.01). Conversely, testosterone significantly reduced the relative *AR* mRNA expression levels compared with the control during differentiation of primary myoblasts (*P* < 0.01). These data suggested that testosterone might induce the expression of *AR* in adipocytes and decrease *AR* expression in skeletal muscle, which were consistent with the previous *in vivo* experimental results. However, the trends of *AR* mRNA expression were broken when EPI-001 was added (*P* < 0.01), especially in EPI-001 and testosterone+EPI-001 group (Figure 8F). Meanwhile, testosterone treatment induced relative *TFAM* mRNA expression levels compared with the control during adipogenesis (*P* < 0.01), accompanied by a decrease in myogenesis at 4 and 6 days (*P* < 0.01) (Figure 8G). The same trend between *AR* and *TFAM* mRNA expression indicated that *TFAM* may be positively regulated by *AR*. Furthermore, the correlation analysis of *AR* and *TFAM* indicated that both adipogenesis and myogenesis showed a significantly positive correlation between *AR* and *TFAM* (*r* = 0.968, *P* < 0.01; *r* = 0.757; *P* < 0.01) in testosterone (-) and EPI-001 (-), and so did testosterone (+) and EPI-001 (-) (*r* = 0.922, *P* < 0.01; *r* = 0.855; *P* < 0.01). This correlation decreased in testosterone+EPI-001 treatment (*r* = 0.384, *P* > 0.05; *r* = 0.223, *P* > 0.05) (Figure 8H).

**FIGURE 9:**
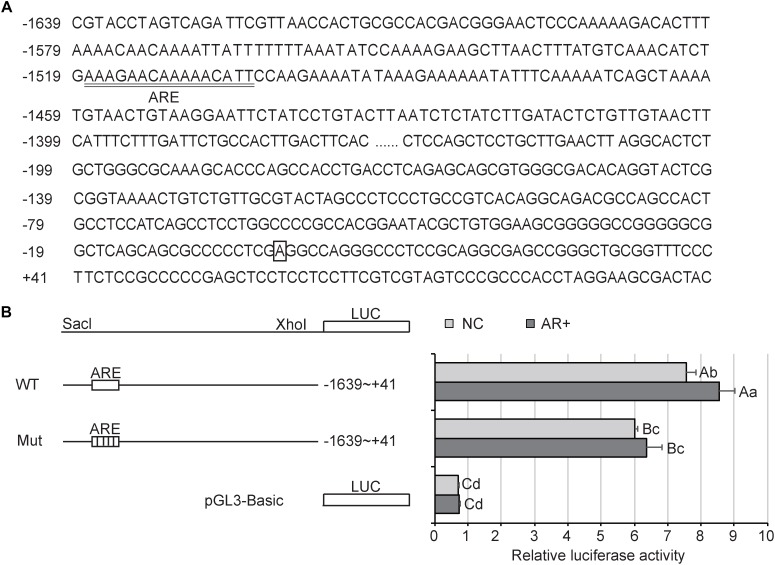
Activity analysis of AR in PK15 cells. **(A)** Sequence of the *TFAM* promoter. Nucleotide positions are indicated on the left side, with the transcriptional start site designated +1 and boxed. The putative AR elements (ARE) are underlined. **(B)** *TFAM* reporter and luciferase assays in PK15 cells. Left: schematic representations of the luciferase construct driven by the wild-type (WT) *TFAM* promoter with putative AR binding sites (ARE) or mutant (Mut) reporter with mutated ARE sites. Right: luciferase activity of PK15 cells transfected with the indicated reporter. “NC” represented “negative control” that AR is not overexpressed; “AR+” presented AR is overexpressed. All data are expressed as means ± SD, 4 technical duplication in each group. Statistical comparisons were performed with Student’s *t*-test with Tukey’s *post hoc* test. The same letters indicate no significant difference (*P* > 0.05); the data shown with different lowercase letters indicate a significant difference (*P* < 0.05); the data shown with different capital letters indicate a significant difference (*P* < 0.01).

*In silico* analysis (JASPAR and PROMO) of the *TFAM* promoter predicted the presence of AREs in the -1503 to -1517 region (Figure 9A). Thus, we performed luciferase assays using a reporter vector, driven by the *TFAM* promoter, containing the putative AREs or a mutated AREs. As shown in Figure 9B, where AR was not overexpressed, the activity of the wild-type reporter was approximately 11-fold higher than the PGL3-Basic (*P* < 0.01). However, the mutant reporter showed a notably lower activity compared with the wild-type, of about 0.80 (*P* < 0.01). Further, overexpression of *AR* significantly promoted the activity of the wild-type reporter (*P* < 0.05), instead of the mutant one. These results indicated that AR drove activation of the *TFAM* promoter, and this upregulation may require binding through the AREs in the TFAM promoter region.

Taken together, these findings demonstrated that testosterone might be involved in the pathway linking *PGC1α* to mitochondrial biogenesis, through AR interacting with the *TFAM* promoter.

## Conclusion

This study provided a relatively detailed tissue expression profile of mtDNA copy number and *AR* expression. We found that testosterone deficiency in a castration model modulated mitochondrial biogenesis through the *PGC1α*/*TFAM* pathway. We also found that the mRNA expression of *AR* was significantly correlated with *TFAM* and our results suggested that *AR* regulates mitochondrial biogenesis by acting on *TFAM*. Overall, our data showed that *TFAM* is an *AR* target gene and involved in the pathway linking *PGC1α* to mitochondrial biogenesis. Of course, our study still has some shortcomings, such as the cell heterogeneity in tissue and the corresponding dilution effect on gene expression, but it is difficult to avoid these problems with current technology. We believe that these problems will be solved with the large-scale application of single cell sequencing in the future.

## Author Contributions

CL, JM, and JZ designed the experiments and wrote the manuscript. HZ and YZ carried out the experiments data organization and statistical analyses. LZ, YJ, GT, XL, and ML provided the experimental samples. JQ and LL participated in the study design and discussed the manuscript. All authors read and approved the final manuscript.

## Conflict of Interest Statement

The authors declare that the research was conducted in the absence of any commercial or financial relationships that could be construed as a potential conflict of interest.
