# Supplementary material for: Testosterone Deficiency Caused by Castration Modulates Mitochondrial Biogenesis Through the AR/PGC1α/TFAM Pathway
Source: Front Genet. 2019 May 29;10:505. doi: 10.3389/fgene.2019.00505 (PMC6548818; doi:10.3389/fgene.2019.00505)
Supplement: Supplementary file 1 [file Data_Sheet_1.docx]

Table S1. Primers used in RT-qPCR analysis

| Species | Gene symbol | Primers (5′-3′) | Production size |
| --- | --- | --- | --- |
| Sus scrofa | *ATP6*  *COX1*  *ND1*  *GCG*  *PGC1α*  *Tfam*  *PRKN*  *Bnip3*  *Pink1*  *ATG7*  *AR*  *GAPDH*  *β-actin* | F: TATTTGCCTCTTTCATTGCCC  R: GGATCGAGATTGTGCGGTTAT  F: ACTACTGACAGACCGCAACC  R: TCCAATGGACATTATGGCTC  F: GCCACATCCTCAATCTCCAT  R: GATTAGAGGGTAGGGTATTGGTAG  F: GAATCAACACCATCGGTCAAAT  R: CTCCACCCATAGAATGCCCAGT  F: TTGCGCAGGTCGAATGAAAC  R: CGCAAGCTTCTCTGAGCTTC  F: ACGCTTTCAGGAAGCTAAGGA  R: CGTTTCGCCCAACTTCAACC  F: GGAGAGGAGCAGTACAACCG  R: CCTTACAGTCCCGGCAGAAG  F: ACGGCTCCTGGGTAGAATTG  R: ATCTTCTCCATGTCGCCGTT  F: TGGCTGCTAACGTGCTTCAT  R: GCGCCTCATACTCCAGGTTG  F: TTCCTGTCAGCCTGGCATTT  R: GGTCTCATCGTCGCTCATGT  F: TGCAGCCTATTGCACGAGAA  R: TCTGGAAAGTCCACGCTCAC  F: CCCCTTCATTGACCTCCACT  R: CCATTTGATGTTGGCGGGAT  F: TCTGGCACCACACCTTCT  R: TGATCTGGGTCATCTTCTCAC | 123 bp  220 bp  99 bp  198 bp  179 bp  182 bp  187 bp  95 bp  195 bp  197 bp  82 bp  157 bp  114 bp |

Table S2. The integrated optical density (IOD) of mtDNA in different tissues

| Tissues | Integrated optical density | |
| --- | --- | --- |
|  | Control | Castration |
| upper layer of backfat  inner layer of backfat  mesenteric adipose  intermuscular adipose  retroperitoneal adipose  greater omentum  psoas major muscle  longissimus dorsi muscle  corpus linguae  Left atrium  left ventricle  adrenal gland  mammary gland  spleen  mesenteric lymph nodes  caecal submucosal lymphatic  liver  lung | 413.17 ± 142.97  403.21 ± 67.34  3739.69 ± 1017.56  1478.17 ± 348.80  1698.26 ± 207.24  1169.61 ± 326.85  10377.05 ± 2642.72  267.06 ± 93.91  8058.11 ± 2683.78  49142.61 ± 6021.38  271735.28 ± 57053.64  69536.86 ± 17524.82  36799.63 ± 20353.65  292.32 ± 119.23  14944.86 ± 7295.49  7902.62 ± 2110.09  24317.95 ± 3887.19  1676.02 ± 481.17 | 871.09 ± 560.34  1114.29 ± 269.63**  3067.77 ± 812.40  9214.42 ± 2534.64**  1614.02 ± 593.74  1799.10 ± 479.62*  5791.74 ± 1696.15**  131.48 ± 37.58**  686.76 ± 219.86**  35351.82 ± 7509.96**  230196.60 ± 36279.70  179595.04 ± 42757.19**  6468.13 ± 3868.87*  173.38 ± 75.45  9585.97 ± 3267.53  10985.48 ± 2653.49*  27153.87 ± 5948.79  1889.46 ± 532.06 |

Single and double asterisks (*, **) indicate statistically significant difference from control group at the 0.05 and 0.01 probability levels, respectively.


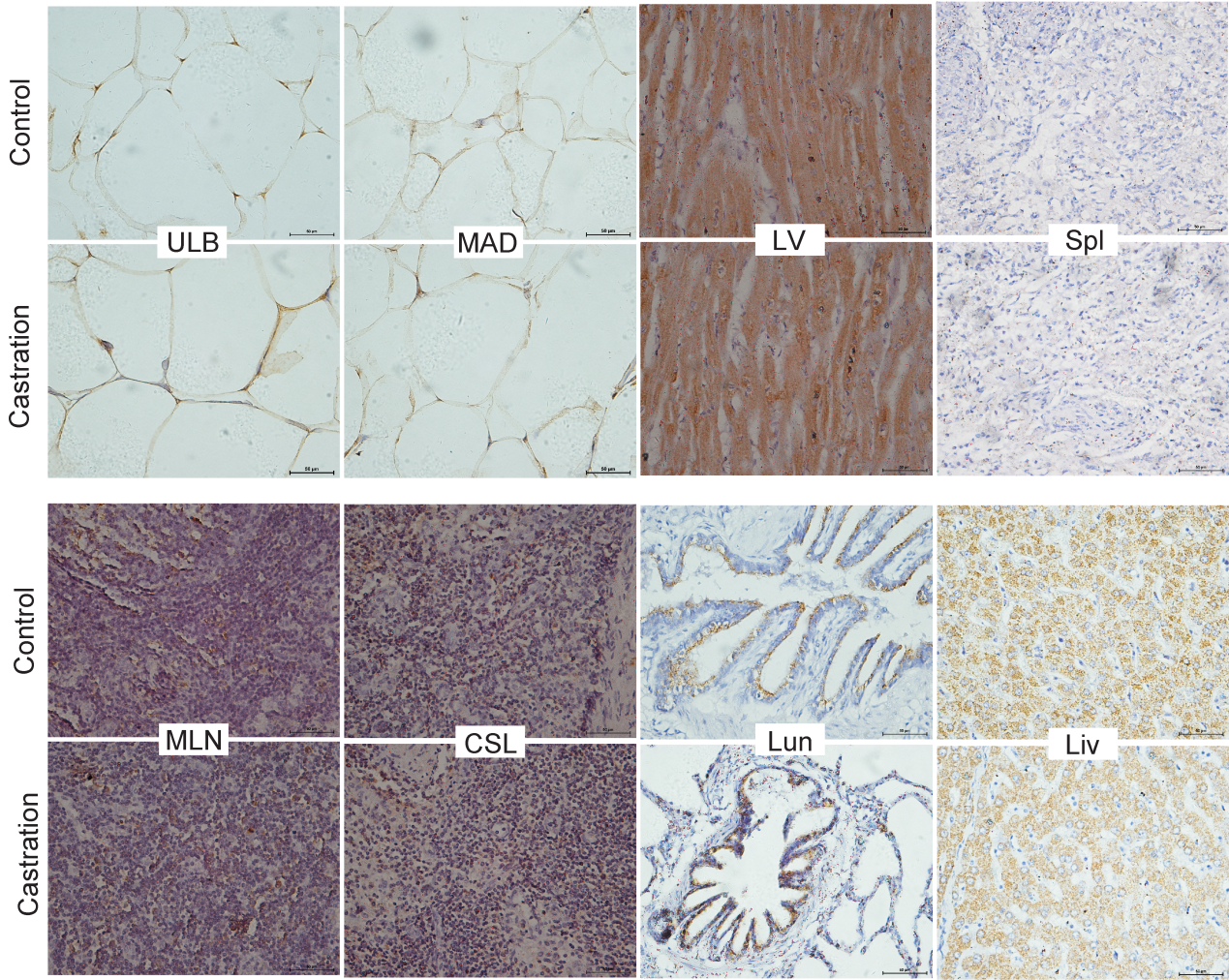


Figure S1. Immunohistochemical analysis for 8 representative tissues using an anti-prohibitin antibody. Scale bars, 50 μm; 400× magnification. ULB, *upper layer of backfat*; MAD, *mesenteric adipose*; LV, *left ventricle* ; Spl, *spleen*; MLN, *mesenteric lymph nodes*; CSL, *caecal submucosal lymphatic*; Lun, *lung*; Liv, *liver*.

Table S3. Correlation analysis of mitochondria-associated genes

| Gene symbol | Control  (correlation coefficient) | Castration  (correlation coefficient) | Fold-change |
| --- | --- | --- | --- |
| *PGC1α* | 0.636 ( *P* < 0. 01 ) | 0.200 ( *P* > 0.05 ) | 3.180 |
| *Tfam* | 0.177 ( *P* > 0.05 ) | -0.264 ( *P* > 0.05 ) | -0.670 |
| *ATG7* | 0.217 ( *P* > 0.05 ) | -0.042 ( *P* > 0.05 ) | -5.167 |
| *PRKN* | 0.360 ( *P* > 0.05 ) | -0.062 ( *P* > 0.05 ) | -5.806 |
| *Pink1* | 0.531 ( *P* < 0. 01 ) | 0.266 ( *P* > 0.05 ) | 1.996 |
| *Bnip3* | 0.487 ( *P* < 0. 05 ) | 0.288 ( *P* > 0.05 ) | 1.691 |

Table S4. Correlation analysis of *AR* and *TFAM*

| Correlation | Control | castration |
| --- | --- | --- |
| *AR*/*TFAM* | 0.388* | 0.390* |

*AR*, Androgen Receptor; *TFAM*, Mitochondrial DNA transcription factor A. Single and double asterisks (*, **) indicate statistically significant difference from control group at the 0.05 and 0.01 probability levels, respectively.

Table S5. The integrated optical density (IOD) of AR in different tissues

| Tissues | Integrated optical density | |
| --- | --- | --- |
|  | Control | Castration |
| upper layer of backfat  inner layer of backfat  mesenteric adipose  intermuscular adipose  retroperitoneal adipose  greater omentum  psoas major muscle  longissimus dorsi muscle  corpus linguae  Left atrium  left ventricle  adrenal gland  mammary gland  spleen  mesenteric lymph nodes  caecal submucosal lymphatic  liver  lung | 31090.86 ± 10229.50  55093.29 ± 14356.25  36740.63 ± 12974.07  77179.37 ± 12046.52  24225.21 ± 3246.78  52424.48 ± 7037.10  88445.89 ± 27140.29  73665.46 ± 19355.96  99717.44 ± 26051.01  155810.92 ± 31382.27  166885.75 ± 25954.53  50058.77 ± 11533.63  5963.55 ± 1439.66  63336.18 ± 20612.07  6978.20 ± 2076.09  113759.03 ± 23896.54  210937.70 ± 36796.49  28243.13 ± 7609.49 | 3368.16 ± 1317.91**  43734.75 ± 11145.12  23477.90 ± 1568.14  106933.25 ± 27318.20*  18838.34 ± 4542.18*  41997.68 ±11249.61  150716.14 ± 28734.97**  120266.76 ± 40707.27*  65138.67 ± 39800.56  233521.83 ± 35875.89**  195230.20 ±15021.17*  23709.69 ± 8674.78**  40189.88 ± 13062.57**  22977.89 ± 6552.40**  10417.63 ± 3839.61  152804.90 ±11479.49**  144661.46 ± 42740.41*  52911.26 ±17025.11** |

Single and double asterisks (*, **) indicate statistically significant difference from control group at the 0.05 and 0.01 probability levels, respectively.


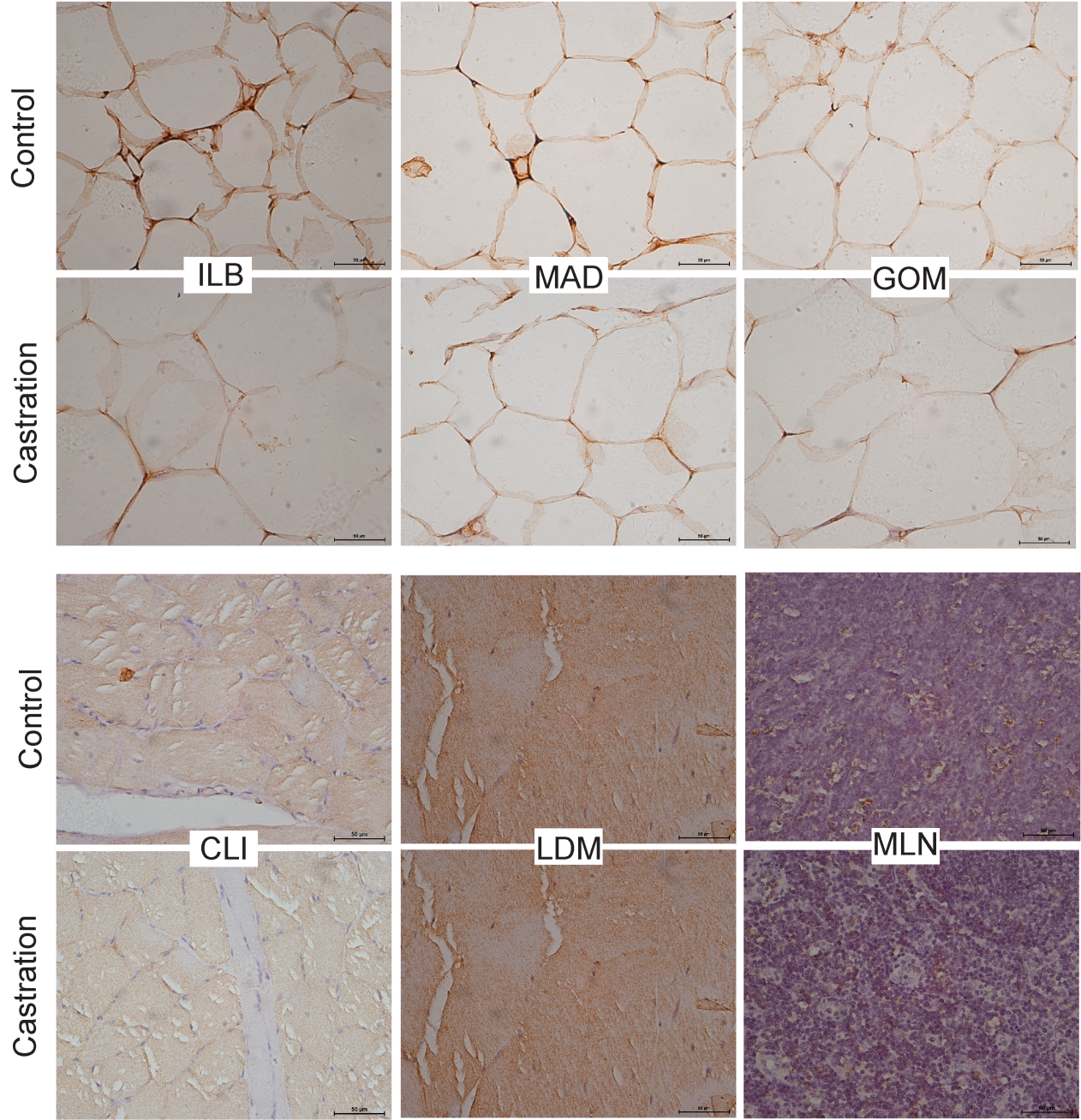


Figure S2. Immunohistochemical analysis for 6 representative tissues using an AR (C-19) antibody. Scale bars, 50 μm; 400× magnification. ILB, *inner layer of backfat*; MAD, *mesenteric adipose*; GOM, *greater omentum*; CLI, *corpus linguae*; MLN, *mesenteric lymph nodes*.
